# The Use of Non-Prescribed Medicines in Infants from Birth to Six Months in Rural Areas of Polokwane Municipality—Limpopo Province, South Africa

**DOI:** 10.3390/children11040434

**Published:** 2024-04-05

**Authors:** Maishataba Solomon Makwela, Eric Maimela, Makoma Melicca Bopape, Reneilwe Given Mashaba

**Affiliations:** 1 Department of Human Nutrition and Dietetics, Faculty of Health Sciences, University of Limpopo, Polokwane 0727, South Africa; maishataba.makwela@ul.ac.za (M.S.M.); eric.maimela@ul.ac.za (E.M.); makoma.bopape@ul.ac.za (M.M.B.); 2 Department of Public Health, Faculty of Health Sciences, University of Limpopo, Polokwane 0727, South Africa; 3 DIMAMO Population Health Research Centre, University of Limpopo, Polokwane 0727, South Africa

**Keywords:** non-prescribed, medicines, self-medication, exclusive breastfeeding

## Abstract

The WHO and UNICEF recommend that only breastmilk, vitamin drops, oral rehydration solution, and prescribed medicine can go through the infant’s mouth. Non-prescribed medications (NPM) include over-the-counter medications and traditional medicine and are contraindicated during infancy. Furthermore, the updated exclusive breastfeeding (EBF) indicator details that herbal fluids and similar traditional medicines are counted as fluids, and infants who consume these are not exclusively breastfed. However, the use of these items is common among caregivers for various reasons, including religious reasons, cultural beliefs, prevention of diseases, and the treatment of diseases. The practice of administering NPM before six months of age undermines exclusive breastfeeding and can result in undesirable health outcomes. Methods: The purpose of this study was to determine the prevalence of NPM, describe the types of medications used, and explain why caregivers use NMP in infants younger than six months of age. A quantitative approach and a facility-based cross-sectional survey were used to conduct this study. Convenience sampling was used to select clinics, and proportionality and simple random sampling were used to select 146 participants. Data were analyzed using SPSS (29). A *p*-value of *p* < 0.05 was considered significant. Results: In this study, the prevalence of NPM was 75.3%. Of the 146 participants, most were 25–35 years old (54%) and first-time mothers (36.3%). More caregivers had high school and matric (67.1%), and 84.2% of caregivers delivered in public health facilities. Almost three-quarters are unemployed (66.7%) and on a child support grant (56.4%). About 43.6% of infants received NPM within the first month of life. The main source of advice to give NPM was family members (86.4%). The main reasons for administering NPM were the treatment of the umbilical cord (57.3%) and the prevention of colic (32.7%). The results show a statistically significant association between the administration of medication and the age of the infant, *p* < 0.005. Conclusions: Non-prescribed medications are highly prevalent in the rural areas of Polokwane and are practiced by caregivers between the ages of 25–35 years advised by the families. Access to self-medication should be controlled, especially in the first month of life. Interventions to reduce the use of NPM should be targeted at young mothers and their families.

## 1. Introduction

Non-prescribed medications, also known as self-medication, refer to the act of using drugs that have not been prescribed, advised, or regulated by a licensed healthcare professional [1,2,3]. This phrase is applicable when describing individuals who self-diagnose conditions or symptoms and then proceed to use non-prescription medications without seeking advice from a doctor or receiving any form of medical oversight [1,2,3]. It is noteworthy that the exclusion of healthcare providers in medicating infants may compromise the safety and quality of care provided to infants [4]. This is because healthcare providers have specialized training and expertise, which informs their ability to make accurate diagnoses and follow-up care, apply knowledge of drug safety, monitor adverse reactions, and treat illnesses in infants [4]. Infant caregivers are likely not to have such knowledge, which emphasizes the need for a consultative collaboration between the caregivers and the health care providers to make better decisions on the medication to give the infants, especially during the first six months of life. 

Although the prevalence of non-prescribed medicinal use in South African children remains to be fully explored, a study by Bland et al., conducted in South Africa, noted that 97% of infants received non-prescribed medications in the first three months of life [5]. These medications were mostly traditional and were given to alleviate perceived constipation and/or colic symptoms [5]. Moreover, research conducted by Friend-du Preez et al. documented that 72.8% of non-prescribed drugs administered to children under 6 months of age were traditional, and the reasons for providing the medicines included parental doubt of the efficacy of Western medicine, preference, and solving perceived supernatural problems [6]. In addition, a study conducted in Nigeria reported a non-prescribed medicine prevalence of 47.7%, while Edessa et al. reported a prevalence of 45% in low to middle-income countries [7,8]. Thus, the prevalence of non-prescribed medications remains high in South Africa compared to other African and low to middle-income countries, inspiring investigations aimed at uncovering site-specific factors that contribute to this high prevalence in rural South Africa.

Given that non-prescribed medications are administered without consulting medical professionals, they may have severe side effects on children, compromising infant health, causing delay in growth and development, and likely causing mortality [9,10,11,12]. However, the inclusion of non-prescribed medicine may not affect exclusive breastfeeding; depending on the type of medicine, quantity, and method of administration, administering non-prescribed medicine to infants presents a risk due to the lack of guidance from certified health professionals. Firstly, without proper medical guidance and advice, caregivers may struggle to give the correct dosage, leading to overdosing and risk of toxicity and potential development of antibody resistance with subsequent adverse reactions [13,14,15]. Secondly, given that this medication is given on the basis of self-diagnosis, this comes with the risk of delaying the real diagnosis for the infant, especially if the non-prescribed medicine is perceived to offer temporal relief to the symptoms.

The World Health Organization advises feeding infants only breast milk for the initial 6 months of their lives [16]. Exclusive breastfeeding involves providing infants with only breast milk for the initial 6 months, excluding additional liquids such as water and solid foods, except for oral rehydration solution or prescribed drops/syrups of vitamins, minerals, or medications recommended by a qualified healthcare provider [16,17,18]. The provision of additional fluids and/or solids has been reported to interfere with exclusive breastfeeding. Furthermore, the updated exclusive breastfeeding indicator details that herbal fluids and similar traditional medicines are counted as fluids, and infants who consume these are not exclusively breastfed [19]. This interference was reported in several studies and was influenced by lack of knowledge, household responsibilities, and the mother’s perceptions of lack of milk or milk not being enough for the baby [17,20]. Despite the WHO recommendation, the utilization of non-prescribed, over-the-counter self-medication, enemas, and traditional medicines is on the rise in South Africa across all cultural backgrounds. Depending on the type of non-prescribed medicine (over-the-counter, traditional, or home-made), method of preparation and administration (drops, mixed with water or soft porridge), and quantity of medicine given to the infant, exclusive breastfeeding may be disturbed, resulting in predominant breastfeeding as opposed to exclusive breastfeeding. 

Evidence from studies that evaluated the effect of exclusive breastfeeding compared to predominant or partial breastfeeding in the first month of life suggested that exclusive breastfeeding reduces mortality rates, infection-related mortality, risk of sepsis or other infections, and respiratory tract infections [21]. Furthermore, exclusive breastfeeding has been associated with enduring advantages, such as lowering the likelihood of excess weight and obesity during childhood and extending into adolescence [22]. Given the sub-optimal prevalence of exclusive breastfeeding in South Africa (31.6%) [23] and the Limpopo province and the study area (39–43.6%) [17,24] compared to WHO goals of achieving a 50% rate of exclusive breastfeeding by 2025 [25], the exploration of the use of non-prescribed drugs, especially in areas with low socioeconomic status remains of important. There are several reports on the use of non-prescribed medicines among lactating women. However, literature related to the use of non-prescribed medicines among lactating children below six months is almost nonexistent. The findings of this study may help policymakers design policies related to improved child health. Therefore, this study aims to explore the use and extent of non-prescribed medicine in children under the age of 6 months.

## 2. Methodology

### 2.1. Research Design and Setting

This study utilized a quantitative approach and cross-sectional design to examine the usage patterns of non-prescription medications among caregivers of infants aged from birth to six months in rural clinics located within the Polokwane municipality of the Limpopo Province, South Africa. Considering that the cross-sectional study design involves collecting data at a specific moment and that it is particularly useful for swiftly and inexpensively assessing disease prevalence or characteristics [26], it was deemed suitable for the current study, given the constraints of data collection timelines. The clinics were selected on the basis that they offer comprehensive health services, including antenatal, postnatal, and child health services, to local communities and the general public. The area is dominated by Sepedi-speaking people, with a minority of people who speak Xitsonga, Tshivenda, and Shona, who prefer to speak in English. During the clinic visit, caregivers receive health talks on various health topics, including infant and young child feeding, offered by health professionals and community health workers. 

### 2.2. Population and Sampling of the Study

Caregivers of infants aged 0 to 6 months who received primary health care services in clinics were the target population for this study. The term caregivers refers to mothers and guardians who are responsible for caring for the infants. In this study, five clinics were conveniently sampled based on their accessibility to the researcher. At the time of data collection, records from the clinics indicated that roughly 240 children, aged 0 to 6 months, were receiving post-natal care across the five clinics combined, resulting in an estimated population of caregiver-infant pairs of about 240. 

This population size was used to calculate the sample size for the present study using the Krejcie and Morgan formula, and the calculations were reported elsewhere [17]. Upon calculating the sample size, it was determined that approximately 146 caregiver-infant pairs would be the minimum required for the present study. Proportionality sampling was applied to determine the number of participants from each clinic, which was approximately 30 participants per clinic. The study took place between September 2018 and January 2019. Participants were recruited and randomly selected. Participants who agreed to participate were given individual appointment dates if they could not participate on the date of recruitment. The names of eligible participants obtained from the clinic register were listed in numerical order. The listed names were randomized using an Excel software 2016 random function, and the sample was obtained by counting the required proportion of participants from top to bottom of the list until the required sample size was reached. The researcher visited the facilities on specific days that the selected participants were scheduled for child immunization. The recruitment was carried out, and those who gave their consent were interviewed. In the event that a participant did not attend the clinic on the day of data collection, the next person on the list was picked up and interviewed. Sepedi is the dominant language spoken in the study area, followed by English. Consent and interviews were conducted using the local language, and English was applicable. Only participants who were at least 18 years old were included in the survey because they could consent to and provide the required information. 

### 2.3. Instruments and Data Collection

Data were collected using a validated closed-ended questionnaire adapted from Goosen (2014) [27], which consisted of two sections. The demographic profile and infant feeding information included medical information. The demographic profile questions focused on caregiver and infant profiles, while infant feeding information focused on infant feeding knowledge, infant feeding practices, and medical information. The data collection instrument was validated through content and face validity and piloted. The content validity of the instrument was ensured by comments provided by the two supervisors of this study and nutrition lecturers at the University of Limpopo. Dietitians who worked in the clinics provided facial validity. The questionnaire was available in English and translated into Sepedi, which is the primary language spoken in Polokwane. 

A researcher-administered questionnaire was used to interview all consenting caregivers who met the inclusion criteria. A rehearsal/mini-training of the interviews was conducted with the supervisors prior to the pilot study. The researcher/interviewer’s bias was minimized by adhering to the questions in the questionnaire and minimizing the use of his own words. To ensure consistency of data collection across different interviews, a similar questionnaire was used throughout the study, and the researcher, who is a qualified dietitian, was the only person who collected the data. 

### 2.4. Data Analysis

The Statistical Package for Social Sciences software (SPSS) version 29 was used for the data analysis. The distribution of variables was determined. The chi-square test was used to determine the association between variables and the confidence level set at a 95% confidence interval. The *p*-value of 0.05 was regarded as statistically significant. Participants with missing data were excluded from the analysis. 

### 2.5. Ethical Issues

The Turfloop Research Ethics Committee (TREC) of the University of Limpopo granted the approval, with the clearance certificate number TREC/117/2017: PG. All participants in this study signed a written informed consent form. Participants were informed of their autonomy to leave the study at any time without any negative outcome and were informed that participation was completely voluntary. The personal information of the participants was kept in a secure place where only the researcher and supervisors could access it. In addition, permission was obtained from the Limpopo Province Department of Health in South Africa to conduct this study.

## 3. Results 

### Socio-Demographic Profile

The present study constituted one hundred and forty-six (146) pairs of mothers and infants with a response rate of about 98.6%. Table 1 shows that most of the mothers were in the age group 26–35 years (54.1%), followed by those in the age group 19–25 years (27%), age > 35 years (13%), with those in the age group of <18 years accounting for 5.55%. The majority of the participants were Sepedi-speaking (84.9%), with the remaining 15.1% being English speakers. About 67% of the caregivers had a high school qualification, 31.5% had received tertiary qualification/education, and about 1.4% of the participants had a primary school educational level. The present study found a high level of unemployment (66%) among caregivers. Most infants included in the study were aged 4–6 months old (49.3%), followed by those aged 2–3 months old (45.9%) at the time of data collection. Only 4.8% of infants were aged less than one month old at the time of data collection. About 51.4% of the infants were boys. Most caregivers in the present study were first–time mothers (36.3%), followed by second-time mothers at 30.1%, with third and fourth-time mothers accounting for 17.8% and 15.8%, respectively. Over half of the participants (53.4%) of caregivers received child support grand. Most infants were delivered in public health facilities (84%), followed by those born in private hospitals, accounting for 14.4%, and those born at home, accounting for 1.4%. 

**Figure 1:**
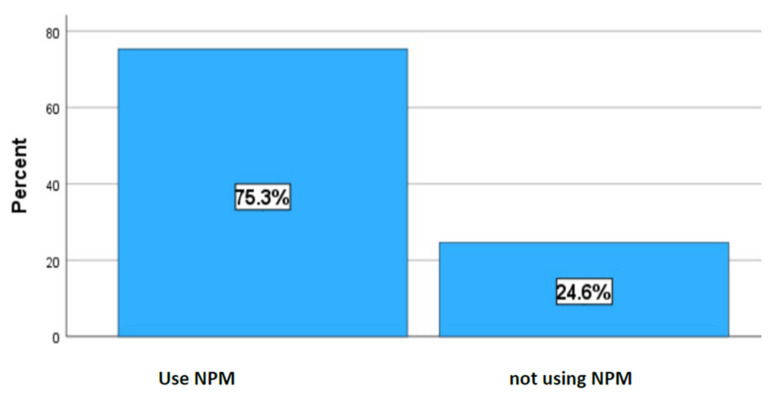
The use of non-prescribed medicine by caregivers.

Figure 1 represents the prevalence of non-prescribed medicine. In this study, the prevalence of the administration of non-prescribed medications by caregivers on infants was 75.3% (Figure 1).

In the current study, a statistically significant association was observed between the use of non-prescribed medicine and the age of the infant (*p* < 0.001), Table 2. There was no significant association between the use of non-prescribed medicine and the language spoken by caregivers (*p* = 0.601), the level of education (*p* = 0.116), the age of the mother (*p* = 0.276), the employment status (*p* = 0.709), the order of birth of the infant (0.653), the support grant (*p* = 0.213), the gender of the infant (0.336). Caregivers with high school levels had a higher prevalence (70.9%) of giving non-prescribed medicine compared to 27.3% of those with tertiary education. First-time caregivers had a higher prevalence (36%) of giving drugs compared to caregivers who had two or more children. Almost 60% of caregivers who received a child support grant administered medicine to their babies before 6 months. In terms of the sex of the infants, there is no difference in receiving non-prescribed medicine from the caregivers. More babies (53.6%) received medications between 4 and 6 months, followed by 2 and 3 months (45.5%) at the time of data collection, and the association between them was statistically significant (*p* < 0.001).

**Figure 2:**
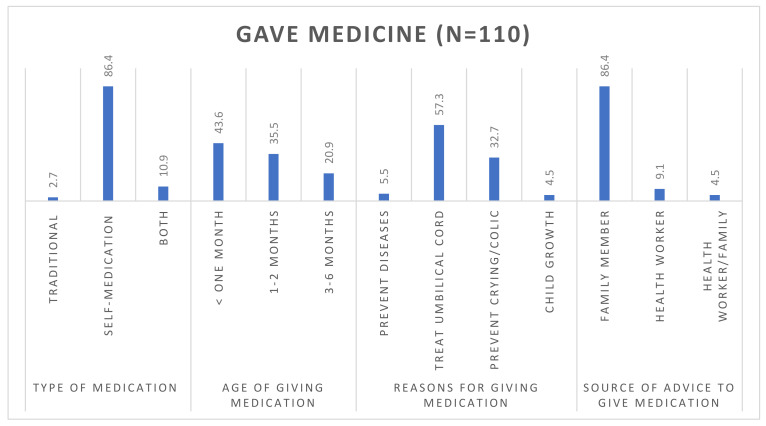
Medical information and the use of non-prescribed medications.

Figure 2 illustrates sources of advice for using non-prescribed medication. Of the 146 caregivers, 75% (n = 110) administered non-prescribed medications to babies less than six months of age. Of those who gave non-prescribed, 86% of caregivers administered self-prescribed medicines to infants below one month (43.6%). The main reasons for administering non-prescribed medicine were umbilical cord treatment (57.2%), prevention of colic (32.7), prevention of diseases (5.5), and child growth (5.4), as advised by family members (86.4%) (Figure 2).

## 4. Discussion

This study aimed to describe the use of non-prescribed in infants before the age of 6 months in Polokwane, South Africa. A total of 146, with approximately 30 participants per clinic, took part in the present study. In this study, a high prevalence of 75.3% of caregivers was found to have given their infants non-prescribed medicines, with only 24.75 refrained. The fact that only 24.75% of caregivers refrained from giving non-prescribed medicines to their infants indicates a concerning pattern of behavior that warrants attention and further investigation, given the potential risk associated with non-prescribed medication. The results of the current study align with those of research conducted in several African countries. A study conducted among parents of babies aged under 5 years in South Sudan reported that about 88.3% of mothers practice self–medication on their babies, with the majority of the medications administered being leftover medications from prior treatment [9], potentially putting the babies at risk as medication from previous diagnosis may not work on symptoms of current perceived conditions. Another study conducted in Cameroon reported that about 72.77% of parents of children aged less than 5 years gave non-prescribed medicine, with a high percentage of those medications being sourced from street sellers [28]. Although these studies reported a high prevalence of the use of non-prescribed medicine in children, they were conducted in children under 5, which is a different age group than the present study. 

Contrary to the findings of the present study, a study conducted in Brazil by Santos DB et al. reported contrasting results in which 59.7% of children had taken medications that had not been prescribed by a doctor, while a prevalence of non-prescribed medicine of 63.5% was reported in Turkey [29,30]. On the other hand, a study conducted in Seshego, a peri-urban location in Limpopo province, South Africa, reported that 47.3% of caregivers used non-prescribed medications in children, while a study in Soweto, South Africa, found 16.4% prevalence of non-prescribed medicine use in under six, respectively [30]. Additionally, a study in KwaZulu-Natal reported that 97% of infants received non-prescribed medications in the first 3 months of life: 98 (89%) rectally and 64 (58%) orally [1]. It is interesting to note that the prevalence of non-prescribed medicine varied between different study areas, suggesting that the use of non-prescribed medicine may be influenced by social, economic, and cultural factors related to the area. Furthermore, the difference could be that the earlier studies focused on children aged birth to 5 years, while the current study was on infants below the age of 6 months. Given that the prevalence of self-medication varies according to location, culture, and social norms, it is imperative to educate caregivers about the need for medicines and that, for certain conditions, the baby does not need medication at all. 

Caregivers administer non-prescribed medicine to their infants for a variety of reasons. In the present study, the main reason for giving non-prescribed medicine was for the treatment of the umbilical cord, to prevent diseases, to prevent crying/colic, and to promote the growth of the child. Similar findings were reported in a study conducted in Nigeria, in which it was found that the reasons for non-prescribed medicine administration were related to perceived positive impact on colic and overall improvement of overall infant health [6,7]. Although there are limited studies that reported on the use of non-prescribed medicine for the treatment of the umbilical cord, the present study found that one of the reasons for giving non-prescribed medicine was for the treatment of the umbilical cord. Although treatment of the umbilical cord using non-prescribed medicine may not affect exclusive breastfeeding since the medication can be administered externally, it is worth investigating how it may affect the overall health of the baby, given the prevalence of this practice, especially in African countries. In addition, several studies conducted in South Africa found that caregivers believe that giving Muthiwenyoni and gripe water, which are administered orally, can assist in reducing the pain related to abdominal pain or umbilical treatment, a practice that is contrary to Western medical treatment, which uses topical ointments [5,31]. It is worth noting that this medication can be found over the counter without a prescription and guidance from a licensed medical practitioner to advise on the appropriate dosage and duration of the treatment. A review conducted by Coffey et al. reported that the application of substances to the umbilical cord was carried out in homage to traditions and was intended to promote healing, speed up the separation of the cord, prevent pain, infections, and bleeding, and protect the baby against evil spirits [32]. Given the cultural complexities related to umbilical cord healing/treatment, it is therefore important to investigate culturally appropriate interventions to minimize the use of non-prescribed medicine for this treatment. 

The source of advice on the use of non-prescribed medicine plays a vital role in deciding whether the caregiver will use or not use non-prescribed medicine. In the current study, the main source of advice was family members (86.4%). This may be linked to traditional beliefs, as alluded to above. These belief systems are informed by societal norms, which include the consultation of traditional healers and herbalists for guidance and medication. In addition, familial traditions and rites of passage rituals relating to the infant may also have an impact on the caregiver’s decision to give non-prescribed medication with subsequent disadvantages on exclusive breastfeeding. This sentiment has been reported in a study conducted in KwaZulu-Natal (KZN) province, South Africa, which reported that about 29% of mothers had consulted a traditional healer for issues relating to infant pain [1]. These results are consistent with a systematic review reporting that treatment decisions depend on beliefs about the illness and treatments, availability of treatments, and advice. Furthermore, family influence can also reinforce the reliability of a habitual notion, such as the use of a particular type of medicine within the family [6,30]. Furthermore, the educational level of the mother/caregiver can influence the decision to give non-prescribed medicine. Although not significant, the present study found that highly educated mothers use non-prescribed medicine less compared to those with a lower education level. In contrast, a study conducted in rural KwaZulu-Natal found that more educated women were more likely to administer oral medications [30]. In this study, there was no association between the age of the mother and self-medication. These findings are in contrast to a study in Sudan that found that self-medication was associated with age [9]. These differences could be due to differences in cultures and employment status of caregivers. 

This study did not find any difference in the use of medicines between caregivers who were employed versus those who were unemployed. Furthermore, the most prevalent non-prescribed medicine medicines given in the present study were over-the-counter medicines, followed by a combination of traditional and over-the-counter medication medicines. The results of this study are in contrast to a study conducted in urban areas of Johannesburg, which reports that respondents of higher socioeconomic status are more likely to use over-the-counter medicines and less likely than other groups to use home treatments [30]. The driving force of the use of over-the-counter medicines includes the treatment of fever and cough, parents who consider themselves experienced in treating their children, and avoidance of the high cost of consultation charges [9,29,30]. In contrast, another study found that users of traditional and complementary medicine are more likely to have a low socioeconomic status and that the work status of the mother was the main factor statistically associated with self-medication [30,33]. 

## 5. Conclusions

The administration of non-prescribed medications is highly prevalent in the study area. Family members were found to be the main influence on caregiver’s use of non-prescribed medicines on infants; as such, interventions should be targeted at family members as well. There is an urgent need for parental educational programs and the development of national policies on the use of medicines for an infant from birth to six months for the prevention of morbidity and mortality. Policymakers could strengthen the regulations on commonly used non-prescribed medicine. In addition, the study findings indicate the need for the development and implementation of community education programs targeted at caregivers to address the potential risk associated with using non-prescribed medicine on infants. This program should also target elderly people and traditional healers, who are often the source of information and advice for the use of non-prescribed medication.

## 6. Limitations

This study used closed-ended questions, limiting the responses participants could give, and did not ask for specific names of medicines used. Also, given that the caregivers had to recall the age of the infant at the time they received non-prescribed medicine, this may introduce limitations relating to response bias as well as over or under-reporting. Future studies could conduct qualitative studies to ask questions that should cover the names of the medicines administered and the reason for giving such a medicine. The data was collected from a small sample; therefore, the findings of this study cannot be generalized to people living in the municipality of Polokwane. Future research exploring other rural contexts of non-prescribed medicine use should be undertaken. 

## Figures and Tables

**Table 1:** Sociodemographic information participants.

Variables	Categories	n = 146 (%)
Age of mother/caregiver	<18 years	8 (5.5)
	19–25 years	40 (27.4)
	26–35 years	79 (54.1)
	>35 years	19 (13.0)
language	Sepedi	124 (84.9)
	English	22 (15.1)
Level of education	primary school	2 (1.4)
	high school/matric	98 (67.1)
	tertiary education	46 (31.5)
Employment status	Employed	49 (33.6)
	Unemployed	97 (66.4)
Age of infant at time of data collection	Birth–1 month	7 (4.8)
	2–3 months	67 (45.9)
	4–6 months	72 (49.3)
Gender of infant	Boy	75 (51.4)
	Girl	71 (48.6)
Birth order	First born	53 (36.3)
	Second born	44 (30.1)
	Third born	26 (17.8)
	Fourth and more	23 (15.8)
Receiving support grant	Yes	78 (53.4)
	No	68 (46.6)
Place of birth	public hospital/clinic	123 (84.2)
	private hospital/clinic	21 (14.4)
	Home	2 (1.4)

**Table 2:** Association between sociodemographic information and the use of non-prescribed medicine.

Variables	Response Categories	Total = 146	Use of Non-Prescribed Medication		
			Gave Medicine (n = 110)	Did Not Give Medicine (n = 36)	*p*-Values
Caregiver’s language	Sepedi	124	93 (84.5)	31 (86.1)	0.601
	English	22	17 (15.5)	5 (13.9)	
Level of education	Primary school	2	2 (1.8)	0 (0.0)	0.126
	High school/matric	98	78 (70.9)	20 (56.0)	
	Tertiary education	46	30 (27.3)	16 (44.4)	
Age of caregiver in years	<18	8	8 (7.3)	0 (0.0)	0.276
	19–25	40	28 (25.5)	12 (33.3)	
	26–35	79	61 (55.5)	18 (50.0)	
	>35	19	13 (11.8)	6 (16.7)	
Employment status	Employed	110	36 (32.7)	74 (67.3)	0.709
	Unemployed	36	13 (36.1)	23 (63.9)	
Infant’s birth order	1st	53	40 (36.4)	13 (36.1)	0.653
	2nd	44	34 (30.9)	10 (27.8)	
	3rd	26	21 (19.1)	5 (13.9)	
	4th	23	15 (13.6))	8 (22.2)	
Support grant	Yes	110	62 (56.4)	48 (43.6)	0.213
	No	36	16 (44.4)	20 (56.6)	
Infant’s gender	Boy	75	54 (49.1	21 (58.3)	0.336
	Girl	71	56 (50.9)	15 (41.7)	
Age of infant at time of data collection	One month and less	7	1 (0.9)	6 (16.7)	<0.001
	2–3 months	67	50 (45.5)	17 (47.2)	
	4–6 months	72	59 (53.6)	13 (36.1)	

## Data Availability

This article utilizes data obtained from caregivers of infants aged 0 to 6 months in the Polokwane municipality, Limpopo province, South Africa. The data presented in this study are available on request from the corresponding author due to privacy and ethical requirements.
